# Diagnostic layering: Patient accounts of breast cancer classification in the molecular era

**DOI:** 10.1016/j.socscimed.2021.113965

**Published:** 2021-06

**Authors:** Emily Ross, Julia Swallow, Anne Kerr, Choon Key Chekar, Sarah Cunningham-Burley

**Affiliations:** a Usher Institute, Old Medical School, University of Edinburgh, Edinburgh, Scotland, EH8 9AG, UK; b Department of Sociological Studies, University of Sheffield, Elmfield Building, Northumberland Road, Sheffield, S10 2TU, UK; c Centre for Biomedicine, Self and Society, Usher Institute, Old Medical School, University of Edinburgh, Edinburgh, Scotland, EH8 9AG, UK; d School of Social and Political Sciences, University of Glasgow, Glasgow, Scotland, G12 8QQ, UK; e Division of Health Research, Faculty of Health & Medicine, Lancaster University, Lancaster, LA1 4YG, UK

**Keywords:** Breast cancer, Genomic medicine, Diagnosis, Qualitative research, Gene expression profiling, UK

## Abstract

Social scientific work has considered the promise of genomic medicine to transform healthcare by personalising treatment. However, little qualitative research attends to already well-established molecular techniques in routine care. In this article we consider women's experiences of routine breast cancer diagnosis in the UK NHS. We attend to patient accounts of the techniques used to subtype breast cancer and guide individual treatment. We introduce the concept of ‘diagnostic layering’ to make sense of how the range of clinical techniques used to classify breast cancer shape patient experiences of diagnosis. The process of diagnostic layering, whereby various levels of diagnostic information are received by patients over time, can render diagnosis as incomplete and subject to change. In the example of early breast cancer, progressive layers of diagnostic information are closely tied to chemotherapy recommendations. In recent years a genomic test, gene expression profiling, has become introduced into routine care. Because gene expression profiling could indicate a treatment recommendation where standard tools had failed, the technique could represent a ‘final layer’ of diagnosis for some patients. However, the test could also invalidate previous understandings of the cancer, require additional interpretation and further prolong the diagnostic process. This research contributes to the sociology of diagnosis by outlining how practices of cancer subtyping shape patient experiences of breast cancer. We add to social scientific work attending to the complexities of molecular and genomic techniques by considering the blurring of diagnostic and therapeutic activities from a patient perspective.

## Highlights

• Molecular techniques are embedded within contemporary UK breast cancer care.
• Routine diagnostic practices ‘split’ breast cancer into subtypes.
• The diversity of diagnostic techniques can render diagnosis both partial and subject to change.
• We capture these experiences from a patient perspective with the concept of ‘diagnostic layering’.
• Gene expression profiling could finalise, but also prolong, diagnostic processes.

## 1 Introduction

Genomic medicine, which draws on DNA sequencing technology to predict disease and ‘personalise’ treatment, is often represented as poised to transform the delivery of healthcare (Samuel and Farsides, 2017; Tutton, 2014). However, social scientists and historians have traced how wider practices of molecular medicine, of which genomics is a part, have slowly been reconfiguring oncology as a specialism since the mid-20th century. This has entailed and provoked the embedding of research within clinical practice (Keating and Cambrosio, 2012), redefinitions of professional roles (Bourret et al., 2011), and has reshaped understandings of cancer itself (Bell, 2013). Alongside attention to the role of hormones and single genes in cancer, the identification of tumour markers has led to the development of ‘targeted’ therapies. This scientific and clinical work is entwined with practices of classification as cancers become divided into distinct subtypes, in some cases according to whether or not they respond to particular therapies (Keating et al., 2016).

The success of molecular approaches has been particularly apparent in breast cancer. Since the 1990s three cellular receptors, Estrogen (ER), Progesterone (PR) and human epidermal growth factor (HER2) have been used to classify breast cancer into subtypes. The development of hormone treatments and a targeted therapy, Herceptin, has led to defined treatment pathways for individual patients where they test positively for ER, PR and HER2 status (Keating et al., 2016). Molecular subtyping has developed rapidly alongside newer genomic techniques. As a consequence, breast cancer is becoming increasingly heterogeneous as it is further divided into categories of ‘basal-like’ and ‘luminal’ cancers, and according to known single gene markers such as BRCA1/2 (*ibid*). Indeed, a recent article in *Nature*'s *Breast Cancer* journal suggests that this (ongoing) fragmentation demands that breast cancers be considered as rare diseases (Bartlett and Parelukar, 2017).

Reflective of its heterogeneity, in the UK today a diagnosis of breast cancer is multifaceted, informed by factors including the presentation of symptoms, clinical imaging and laboratory analysis of tumour tissue. Pathology examinations occur at several time points, following one or more biopsies and surgeries to remove the cancerous and surrounding tissue. Alongside histological laboratory work to establish characteristics such as the tumour's size and whether it has spread to the lymph nodes (LN), the examination of the ER, PR and HER2 status of tumour tissue to determine molecular subtype is a routine aspect of breast cancer diagnosis in the UK. This provides an indication of the suitability of hormone treatments and Herceptin for each patient (Yeo et al., 2014). In addition to these well-established molecular practices, genomic approaches are also becoming implemented within routine NHS diagnostic processes. The hope attached to these techniques is that they will lead to a more precise understanding of cancer type, and better guide prognosis and treatment decision-making for individual patients.

In 2013 a genomic technique, gene expression profiling, was approved for use within the UK NHS for a defined group of patients with early stage ER/PR+, HER2-and LN- breast cancer (National Institute for Health and Care Excellence, 2018). This cancer type is highly responsive to hormone treatment and associated with a good prognosis, with some patients avoiding adjuvant chemotherapy. The stratification of this group of patients into those who will and will not receive chemotherapy is emblematic of precision medicine. In the UK today this is aided by prognostic algorithms, which draw on information about the patient and their tumour markers to calculate an estimation of chemotherapy benefit. However, for a small proportion of these patients, a recommendation for chemotherapy can remain elusive following use of these tools. It is at this point that gene expression profiling may be requested by NHS clinicians.

The first gene expression profiling platform approved for NHS use in the UK was the US-developed Onco*type* DX test. This test analyses 21 genes in tumour tissue taken at surgery to develop prognostic information (the likelihood of cancer recurrence), and based on this an estimate of chemotherapy benefit. Today this test has been adopted across the UK NHS to inform chemotherapy decision-making. The addition of gene expression profiling to existing practices of subtyping reflects the fact that clinically and within the laboratory, the number of procedures and tests undertaken on breast tumour samples is increasing. As a consequence, diagnostic processes are becoming more divergent among individual patients (for example, some patients' samples are amenable to further classificatory practices to determine treatment, while for some a treatment recommendation is clear from an earlier stage), and more protracted. An important question for sociologists is how these wider approaches to molecularization for driving more individualised and precise diagnoses impact patients’ experiences of cancer.

Across medical sociology, a number of scholars have considered personal experiences of breast cancer diagnosis (e.g. Liamputtong and Suwankhong, 2015; Sulik, 2009), including of molecular diagnostics through the case of germline BRCA1/2 genetic testing (Hallowell et al., 2004; Hesse-Biber, 2014). Using ethnographic methods, Day et al. (2017) explored patient and practitioner experiences of genomic techniques to stratify treatment within NHS breast cancer care, concluding that in this setting these practices “promoted less rather than more integrated, personalised and seamless care” (p155). Though experiences of breast cancer diagnosis more broadly have often been the focus of social scientific enquiry, less in-depth attention has been given to the well-established processes of diagnostic classification used to identify breast cancer subtypes and guide treatment decision-making. Consideration of routine diagnostic processes in cancer is important because these impact patient interpretations of their disease and treatment decisions, but also because these processes have the potential to become reconfigured with the introduction of genomic techniques.

In this article, we explore women's accounts of being diagnosed with early stage ER/PR+, HER2-and LN- breast cancer, focusing on patients who have experienced Onco*type* DX testing as part of this process. We discuss experiences of well-established clinical diagnostic pathways to identify their specific cancer type, then turn to accounts of the Onco*type* DX test. Contributing to literature on the sociology of diagnosis, we introduce the concept of ‘diagnostic layering’ to make sense of how the multitude of diagnostic processes in oncology, which in the UK today routinely draw on molecular and genomic techniques, shape patients' experiences of being diagnosed with breast cancer. First, we outline some of the sociological approaches to diagnosis that have informed our analysis.

### 1.1 Diagnosis as a process and system of classification

In recent years, the study of diagnosis has become firmly established as a ‘sociology’, with in-depth examination of the procedures and experiences of diagnosis providing insight into the wider social and structural conditions within which it occurs (Jutel, 2015), but also showing how diagnosis acts upon social worlds (Pickersgill, 2014). Sociologists have demonstrated that diagnosis is a both a process shaping patient and professional experience, and also a system of classification working to organise disease and direct treatment (Jutel and Nettleton, 2011). Two fundamental classification practices within medicine are the acts of ‘lumping’, whereby broad categories are created highlighting connections and similarities between conditions, and ‘splitting’, which emphasises specificity and difference (Zerubavel, 1996). Joyce and Jeske (2020) describe the fine-tuning of diagnostic categories through lumping and splitting as an iterative and ongoing process, requiring adjustment as new tests and treatments are developed and new knowledge is produced.

Once established, diagnoses can be acted upon through the establishment of a corresponding prognosis and treatment plan (Jutel, 2009). Diagnostic techniques thus have productive effects within the clinic, with powerful consequences for patients, clinicians and the wider organisation of healthcare practice. However, social scientists have shown that the messiness of disease means medical classifications are uncertain and always ‘configurationally complex’ (Bowker and Star, 1999: 172) as they attempt to bind the “biological, the technological, the social, the political and the lived” (Jutel, 2009: 294) through assemblages of a range of human and non-human actors (see Gardner et al., 2011; Locock et al., 2016).

By tracing diagnosis in practice, existing work has shown that diagnostic categories and procedures can be experienced by patients in varying ways; for example as disembodying in the case of medical technologies (Daly, 1989), but also as empowering, by providing reassurance (Blaxter, 2009) or opportunities to work strategically with diagnostic categories (Joyce and Jeske, 2020). Sociologists have shown that the road to diagnosis is rarely straightforward for individuals and those treating them, often experienced as negotiated (Madden and Sim, 2006), and generative of uncertainty (Timmermans et al., 2017; Swallow, 2020). This has been discussed even in relation to conditions with well-established and apparently stable classificatory procedures. For example, Purkis and Van Mossel (2008) show that in the face of a multitude of technologies and tests, patients navigating the Canadian breast cancer system experienced diagnosis as “neither clear or complete but always in motion, frequently being revised” (p143). The authors found that diagnosis and treatment plans were continuously negotiated, and therefore conceptualise cancer as a contested illness. This characterisation likens cancer to medically unexplained conditions, which can situate patients in a ‘diagnostic limbo’ (Nettleton, 2006) and leave them unable to access treatment - demonstrating the close relationship between these clinical activities (Dumit, 2006).

Genomic techniques have further reconfigured the relationship between diagnosis and treatment, and the wider healthcare systems within which these practices take place (e.g. Bourret et al., 2011; Timmermans et al., 2017). Troubling the sociological delineation of diagnosis as a distinct process, Bourret et al. (2011) have shown how novel molecular techniques can conflate diagnostic, prognostic and therapeutic work. Diagnosis and treatment become intimately connected within molecular approaches, as therapeutic decision-making becomes directly related to knowledge of cancer pathogenesis and progression (Keating and Cambrosio, 2013).

Despite anticipations for genomic techniques to remedy diagnostic uncertainties, particularly in rare genetic diseases where patients seeking a diagnosis often hope these “advanced” tools “may bring their search to an end” (Timmermans et al., 2017: 442), their mobilisation does not always provide a resolution. These techniques can produce uncertain results and interpretive dilemmas for clinical teams (Skinner et al., 2016). This has also been discussed in relation to efforts to mainstream genomic technologies into routine care. Here the fallibilities of novel techniques, and a lack of capacity to interpret complex genomic information, can demand careful work from healthcare practitioners to manage patients’ expectations for treatment (Kerr et al., 2019).

In this article, we draw on qualitative interview and observational data to explore these issues in the context of routine cancer care in a UK setting. We contribute to existing literature by considering the molecularization of breast cancer diagnosis, with specific reference to the identification of cancer markers and gene expression profiling (Onco*type* DX). Drawing on personal accounts we introduce the concept of ‘diagnostic layering’ as a heuristic device to make sense of how these processes of classifying breast cancer shape patient experience. Due to the numerous laboratory analyses now conducted on breast tumour tissue, patients receive information about their cancer in ‘layers’, as separate rounds of clinical information are relayed to them by their clinician. This can be over a period of many weeks. These layers aim towards a more refined diagnosis, as they work to ‘lump’ the suspected tumour as a cancer, and then ‘split’ the cancer into a distinct subtype and subsequently direct treatment. The concept of layering thus entails both of these activities, but additionally captures the temporality of these processes, which occur across various time points and may require repetition. As several layers of diagnostic information from diverse sources are collated to subtype breast cancer today, including histopathological, molecular and genomic analyses, we contend that diagnosis is temporally extended, as well rendered partial (incomplete) and subject to change (mutable). This can impact patients' expectations for diagnostic pathways and care. Despite anticipations for genomic testing to finalise the diagnostic process, patient accounts reveal a more complex picture. We show that the incorporation of these test results into treatment decision-making is not straightforward, but informed by diagnostic information derived from established tools, and powerful cultural imaginaries of cancer and chemotherapy.

## 2 Methods

The data on which this article is based is drawn from a large multi-sited research project considering how genomic techniques in cancer are impacting patient and practitioner experiences of cancer care. As part of this project a range of health professionals were interviewed, with some emphasising the importance of attending not only to novel genomic techniques, but those already established in routine care such as gene expression profiling. As such, following NHS ethical approval (REC reference 16/YH/0229) and with the help of key oncologists and research nurses, we recruited and interviewed 18 patients who had received Onco*type* DX testing as part of their cancer care at NHS sites within England and Scotland. We also conducted four observations of consultations where patients (all with an accompanying family member) discussed treatment decisions in light of Onco*type* DX results. Interviews with oncologists who had used the technique with their patients were also conducted, as well as observation of online discussions of Onco*type* DX testing, with these findings presented elsewhere (Kerr et al., 2021; Ross et al., 2019).

Interviews with patients took place between June 2017–August 2019. Interviews were semi-structured and began by seeking a narrative account of the interviewee's symptoms, suspicions and their path to diagnosis. Interviews also covered engagements with gene expression profiling specifically, including how the technique was explained by their clinician, any independent research they had undertaken about the technique, how they made sense of their Onco*type* DX result, subsequent treatment decision-making and ongoing legacies of the result. Interviews lasted between 45 min to up to 2 h, were audio-recorded and transcribed verbatim. The four observations of consultations took place in June and July 2017. These involved a researcher being present at consultations where treatment decisions were discussed and formalised by patients following their Onco*type* DX result. These lasted up to 1 h. Fieldnotes were taken by hand, before being typed and shared with the research team.

Analysis drew on techniques of constant comparison (cf. Charmaz, 2006) and thematic analysis. Transcripts and fieldnotes were shared between team members, and read and compared in an iterative process moving back and forth between individual transcripts and the complete set of interviews and observations. Similarities and differences were noted within and between transcripts and fieldnotes, with reference to data from the wider project and sociological literature on diagnosis and cancer. Key themes were developed as a result of this process. These included ‘treatment decision-making’ ‘quantification of risk’, ‘embodiment’, and ‘clinical judgement’. The theme of ‘layering’ began as an *in vivo* code that succinctly described participants' experiences of gradually gaining various levels of information about their cancer over time. After going back to the data with this theme in mind, we developed the concept of ‘diagnostic layering’ to convey the ways in which, from a patient perspective, molecular and genomic information shape experiences of contemporary breast cancer diagnosis. We also attended to how processes of diagnostic classification were further shaped for these participants in light of gene expression profiling; in ways that could both end but also extend the diagnostic process.

In what follows, we present women's accounts of ‘diagnostic layering’, and discuss the implications of this for chemotherapy decision-making and wider experiences of cancer. In the first section we attend to how participants discussed well-established routine diagnostic procedures for breast cancer. We share experiences of several of the ‘diagnostic layers’ articulated by patients. These included provisional diagnoses of cancer at the breast clinic, the molecular classification of cancer following pathology results (including informal characterisations of their cancer type by clinicians), and for some the re-casting of their cancers after further pathology. In the second section we attend specifically to personal accounts of Onco*type* DX testing. We show how this aspect of diagnosis became intimately connected to treatment, and related to this, how some women described this as a ‘final’ layer of diagnostic information. However, for others this information could introduce further uncertainties and extend diagnostic processes. We end by considering the implications of genomic techniques for patients' experiences of cancer diagnosis.

## 3 Diagnostic layering in early stage breast cancer

Many participants’ experiences of diagnosis began with an embodied awareness of a lump in their breast, but for a minority this followed a recall after routine screening. At the time of our interviews patients had all been treated surgically, and had commenced or were due to embark on chemotherapy and/or radiotherapy following the results of gene expression profiling.

For most interviewees, the initial labelling of suspicions about a lump or a blur on a mammogram as cancer took place in the breast clinic. The clinic, described by Dette as a “one-stop shop”, provided women with a mammogram, ultrasound and, if necessary, a biopsy on the same day. Valerie described her experience at the clinic as like a “conveyer belt”. The very experience of being in the clinic could serve as a form of diagnosis in itself. Several participants described a number of ‘cues and clues’ that led them to anticipate a diagnosis of cancer before being formally told (cf. Locock et al., 2016). These included being re-approached by the nurse for a second mammogram (Valerie), but also observing the waiting room and noticing other attendees leave after each tier of investigations, whilst they and a dwindling group of women remained (Dette, Eve).

At the end of their visit, which for some lasted the entire day, a discussion with the oncologist at the breast clinic could represent a first layer of diagnosis for participants. Shortly after their final biopsy, a verbal indication of cancer could be communicated to women. For some this was unequivocal, coming as a shock for Bethany who exclaimed “how can she say that when they've not got the biopsy result?“. For others this was implied. Linda was told “don't be surprised” if the pathology report indicated cancer. All were told that more information would be available following the biopsy results, which could take up to two weeks.

### 3.1 Classifying a ‘good’ cancer

Biopsy results provided a further strata of information to participants about their cancer; and in some cases it was not until this point that cancer was confirmed. The pathology report both confirmed a diagnosis of breast cancer (a ‘lumping’ of the tissue's characteristics as ‘cancerous’), but also provided information about the tumour's receptor status e.g. ER+, HER2- (the ‘splitting’ of their cancer into a particular subtype). In describing their diagnosis during interviews, almost all disclosed the receptor status outlined in this first pathology report, and some the size and grade of their tumour, appreciating that this information was specific to their cancer. The heterogeneity of breast cancer was therefore recognised by participants, gleaned not only from their pathology results but also from discussions with clinicians. In two consultations, we observed the oncologist explain to the patient that “breast cancer is a very big family of cancer” (Observation2 July 2017) and “breast cancer is not one cancer” (Observation1 July 2017).

Commonly encountered within interviews were reports of clinicians describing their subtype of the disease as a ‘good’ cancer. This was inferred through explanations of their cancer as “early stages” (Elisa), “treatable” (Lois) and “low risk” (Lillian), or in some cases women's cancers were explicitly described as ‘good’. Eve had attended the breast clinic and was informed that something “sinister” had been found on her ultrasound, leading to several biopsies later that day. During the discussion with the oncologist she was advised of possible scenarios and treatments, but unlike other participants had not been told to expect a diagnosis of cancer. Her next appointment was with a different clinician two weeks later. She recounted:

So when I went in and he said, erm, ‘oh well, it's all good news’. So of course, I was like, ‘oh yes!‘, do you know what I mean?’ And he must've saw my face**,** and he's like, ‘whoa, whoa, what have you been told?’ Right, and I was like, ‘well, basically, in no uncertain terms, I had cancer, but it, the word wasn't used’ … And he went, ‘oh yes, you've got cancer’, he said, ‘but it's a good cancer’.

This way of communicating her first formal diagnosis discursively configures Eve's cancer subtype in a positive way, and as distinct from the widely accepted understanding of cancer more generally as ‘bad news’. A configuration of these women's cancers as good was also achieved beyond linguistic performance. As discussed by Kazimierczak and Skea (2015), this was embedded within a range of socio-material practices and relations (see also Gardner et al., 2011; Locock et al., 2016). For participants in this research, these arrangements comprised their molecular markers which were discussed favourably by clinicians, and as we observed in one consultation, sometimes illustrated visually with pen and paper (Observation2 July 2017). This added to a sense of successful treatment prospects and positive outlook:

When they gave me the results of the oestrogen, the ER-positive, they said that was good because the tamoxifen would be very effective and so the consultant was “that's really good.” **Julie**

This layer of information largely configured our participants' cancers in ways that inspired relief and optimism. Significantly these positive interpretations of their cancer-type impacted upon anticipated treatment pathways, which were again largely discussed optimistically in the first instance, and without mention of chemotherapy. Dette was told in a “matter-of-fact” way that “it's very small, it's very early, you'll find out about radiotherapy but you have to have surgery first.” Similarly Elisa was told “yeah you have got [cancer] but early stages. So we'll just give you surgery then radium”.

Others, however, were given a more tentative outlook, being told that treatment depended upon further factors that would only be resolved following surgery. Valerie was told about her molecular markers in a positive way, but that though appearing favourable there was uncertainty regarding lymph node involvement:

So I was HER2 negative so that helped. Erm, and then obviously whether it was in the lymph nodes or not. From the ultrasound they, they thought it wasn't, but they weren't gonna confirm that until results from the surgery.

Though tentative for several participants, such initial depictions of their cancer as ‘good’, and for some, easily treated, are significant. These impressions paved the way for the shifts in anticipated treatment pathways that were to come, as additional layers of diagnostic information emerged; each achieved through fresh configurations of socio-material practices.

### 3.2 The provisionality of breast cancer diagnosis: reconfigurations of cancer and treatment

As outlined above, initial biopsies taken at the breast clinic gave a degree of diagnostic information about our participants' cancers, and in some cases, indication for treatment. However, further pathology examination following surgery meant that for some, their ‘good’ diagnosis and related anticipations for treatment could be reconfigured.

As in Valerie's extract above, surgery was presented to participants as the point at which uncertainties about treatment could be resolved. However in practice, surgery could result in initial understandings of the tumour being rendered obsolete, and in some cases the designation of their results as a ‘grey area’. This could require further intervention to generate an additional layer of diagnostic information. Some of these uncertainties, notably to do with the cancer's size, could be clinically acted upon. For six of those we interviewed, following breast conserving surgery to remove the cancerous tissue (a lumpectomy) it was discovered that the operation had not removed an adequate margin of cancer-free tissue around the tumour. These six women therefore underwent one or more re-excisions to remove all of the cancerous tissue, and re-establish the size of the tumour.

Other aspects of the cancer were also altered following diagnostic information from surgery, with ‘good’ cancers rendered potentially ‘bad’ cancers. In most instances this was due to confirmation of, or in three cases a shift in, the cancer's grade (rate of growth). This was the case for Lois:

I got the results from the surgeon and that was when he said “oh, your lymph nodes were clear and all the cancer's been removed” so that was a great boost, so that was super. But the cancer had moved from a grade 2 to a grade 3 category so it was worse than first expected.

The transformation of their cancer to a higher grade provoked a shift in language from clinicians. Women reported their doctors now using terms including “aggressive” (Bethany, Dette, Elisa), “high grade” (Zoe), and “fast growing” (Valerie, Eve). In some instances surgery uncovered what was described as a “slight” or “tiniest” spread to lymph nodes, which was the case for Lillian, Jane, Julie and Alice. In situations such as these, where new or altered information about the tumour was introduced, anticipated treatment pathways were (again) rendered uncertain, and chemotherapy could be raised as a possibility where it had not existed before. Due to previous layers of information pointing to a ‘good’ cancer, Elisa, Dette and Lois had not anticipated being offered chemotherapy, with Chrissy also unsure as to why the treatment had suddenly been introduced when “the cancer had all gone from surgery”. Linda had not considered chemotherapy because of heart condition, which had compelled her clinicians to “try and avoid that”. The introduction of chemotherapy came as a “blow” for these women. The instability of diagnosis as related to treatment could work both ways, however, as two participants (Susan and Julie) *had* expected chemotherapy, which was then called into question following surgery.

As well as the initial *incompleteness* of diagnostic information described in the extracts above, which participants (and their clinicians) gained gradually in layers, interviewees therefore also pointed to the *mutability* of diagnosis, particularly following surgical intervention. Subsequent layers of information could both add to and refine existing understandings of the tumour and cancer type, but in some cases called previous layers into question. Paradoxically, though diagnosis is generally engaged upon with a view to fixing a disease classification, and discern treatment along with “a sense of where the road ahead may lead” (Jutel, 2009: 288), for these women the diagnostic process could be experienced as partial and subject to revision, as progressive layers of information invalidated previous knowledge of the tumour, shifted anticipated care pathways, and for some introduced the possibility of a feared treatment. Dette described her experiences of receiving diagnostic information progressively over time as “unsettling”:

When you do ask questions they do say “we can't actually really tell you until we've done this” you know, until they've done the biopsy. And then when they've got the biopsy they say “oh, actually, we're going to do a better test when they actually take the lump out” … I've been told a lot “we don't have a crystal ball”.

Jane experienced diagnosis as a series of “stages”, with waiting for results the most difficult aspect. Hazel explained that thanks to advice from a friend who'd also been treated for breast cancer, she had been prepared for “false horizons” and as such did not take anything in her planned treatment regime for granted. She described being “always open to whatever happens next, without, without holding on too much to what the, the considered next stage ought to be”. Bethany described her experience of receiving contradictory layers of information about her cancer, and the implications of this for treatment, as a “rollercoaster”. However, she also sympathised with this approach:

Being told that because of the Herceptin [HER2] result I didn't need chemotherapy, you know, I felt very positive and I told all my family. Which is why then a few days later, or whenever, I'm told “it's grade three and you probably will”, em, I had to then tell my family that … But I feel that's just the way it has to be because that's just part of the process really. You know, so it's about, sort of maybe eliminating, you know, what it is not, and working out what it is really. And the clinicians have to do that and it takes a wee while.

Bethany's account shows that though it may be subsequently revised, each layer of information had palpable consequences for patients.

The extracts from both Hazel and Bethany point to the significance of diagnostic information not as an end in itself, but with regards what this meant for treatment*.* This was a key issue for many of those we interviewed, who discussed diagnostic details as ‘good’ or ‘bad’ with reference to their implications for chemotherapy. However, for the women in our research, the assessment of diagnostic information through established tools was insufficient to determine a recommended treatment regime, and further layers of clinical information about the cancer were required. In this type of early stage breast cancer, prognosis now came into play. For those we interviewed, prognostic information was derived from an algorithm freely available to clinicians and the public, *NHS Predict*. The women interviewed here were unique, however, in that this layer of information proved inconclusive. For those in our research, estimations of chemotherapy benefit placed women in an ‘intermediate’ range, characterised by many of our participants as a ‘grey area’. A further round of information was sought by their clinicians to refine these estimations, accessed in the form of a genomic technique; Onco*type* DX. Below, we explore how the introduction of Oncot*ype* DX added another ‘layer’ to patients' experiences of diagnosis, in ways that could both finalise, but also extend, the diagnostic process.

## 4 Gene expression profiling

In the context of already protracted experiences of diagnosis, clinical uncertainty around treatment was difficult for participants to come to terms with. Patients had been given a diagnosis of a “scary” disease (Eve) “that kills” (Susan), and which inspired painful recollections of the deaths of friends or family members. Though contemporary medicine has relieved some of the historic devastation caused by the disease and radical treatments (Löwy, 2009), cancer remains culturally feared, in part due to its uncertainties and unpredictability. As described by Jain (2013), the catalogue of explanations, treatments and statistics faced by patients render the disease virtually impossible to navigate. In the face of cancer, interviewees discussed wanting to do all they could to treat and prevent its return, including undergoing chemotherapy. As Eve expressed “I want every treatment I can get, do you know what I mean, to stop it coming back.” However, alongside fears of cancer and its return, many of those we interviewed were equally anxious at the prospect of the treatment, which they associated with hair loss, compromised immunity and long-term side effects (see Bell, 2009). This again included Eve, who described memories of her mother being “ravaged” by chemotherapy.

It is within this context that where a clinician had so far guided them with regards treatment decisions, providing some solace in the face of these oncological uncertainties, unknowns suddenly became openly acknowledged. Further, the responsibility for addressing these could be passed to patients: in the absence of a recommendation from the prognostic algorithm, many interviewees had been asked to choose whether or not to proceed to chemotherapy. Lois described this as her oncologist “laying the decision at your door”, and along with others, experienced this as emotionally fraught. One patient observed in Observation2 July 2017 said that this placed her “between a rock and a hard place”, an idiom also used by Alice who elaborated further: “I didn't want to take a risk of not having chemo if I needed it. But I didn't want to have, take the risk of taking chemo if I didn't need it.” Below, we demonstrate how the layer of information provided by gene expression profiling could both attend to the emotional difficulties of this situation by solidifying a diagnosis and enabling a treatment decision, but also prolong the diagnostic process further by introducing additional contradictions and complexities.

### 4.1 A ‘final layer’ of diagnosis?

Onco*type* DX was represented by clinicians, and understood by patients, as having the potential to resolve clinical uncertainties surrounding treatment. In one consultation we observed the following exchange:

Because the patient is ‘so young’ [early 40s] the consultant predicted a good prognosis. She added that without the Onco*type* test result [i.e. with just the *NHS Predict* result], it might have appeared that the patient doesn't need chemo but with this result it shows the benefit … The consultant said *NHS Predict* gives you ‘a rough average’ but because breast cancer is a big group of diseases, this is ‘where Onco*type* comes in’. **Observation1 July 2017**

Here, gene expression profiling is described as providing a more specific estimation of chemotherapy benefit, contrasting with the ‘rough average’ generated by *NHS Predict*. The notion that gene expression profiling provided a level of refinement not available from other tools was echoed by interviewees. Both Julie and Bethany described the test as providing “more specific” information about their cancer. Bethany, who described Onco*type* DX as a “state of the art” test, and “most advanced that's available” explained this in more detail:

As far as I was concerned, this test, as it had been described to me, meant that this was very particular to me, in a way. You know, this was a sort of DNA profile of my tumour and, to some extent, milder extent, of me and therefore if that test said I needed [chemotherapy] then I would definitely have done it.

Due to the fact that the test generated a recommendation based on analysis of her own tumour tissue, Alice discussed the test as providing “scientific fact”, which she contrasted with the “expertise” of her clinician. Julie noted that the result “will make me feel I'm not making a hunch decision, I'm making a decision based on actual science”. The fact that the test was “personalised”, gave Hazel “confidence” in the subsequent recommendation to forgo chemotherapy. This was echoed by Bethany who, again demonstrating an appreciation of the diversity of breast cancer, explained “it does make me feel more confident that this is about me and em, that attention is being paid to me and the sort of tumour I have”.

Due to its ability to provide a “personalised” recommendation, these interviewees portrayed Onco*type* DX as providing a layer of information to inform their treatment decision which surpassed others. Though previous diagnostic techniques had of course also been personalised, involving molecular analysis and pathological examination of their tumour tissue, treatment recommendations had been more heavily developed from clinical judgement, assisted by tools such as the *NHS Predict* algorithm. Whilst such algorithms and Onco*type* DX perform the same task – estimate recurrence risk to develop a chemotherapy recommendation – the fact that Onco*type* DX's treatment recommendation was directly developed from their “DNA profile”, and thus “personalised”, marked the tool as distinct for some participants, and as inspiring a particular confidence.

For these interviewees, the test result was represented as integral to their decision about chemotherapy. Those with a ‘low risk’ score described their result as allowing them to more confidently say no to chemotherapy:

So by getting the test … then that gave us the second opinion really to say that no, I didn't need the chemo. **Lois**

So I had the test done, so I had the scientific back-up, almost, to say, yeah, you really won't benefit [from chemotherapy]. **Alice**

Similarly, those with a high risk score, despite some being disappointed that chemotherapy had been recommended as a treatment option, also welcomed their Onco*type* DX results. Felicity represented the test as leaving her with no choice but to proceed with the treatment:

I had, well, a score of twenty-seven, which was a no, no-brainer, really. That's, that's you at a high risk so … So in that way that was reassuring. [Laughs]. Do you know, in an awful way. In an awful way. It was like well, I was glad that it was such a definitive result.

Here, Felicity describes the ‘layer’ of diagnostic information provided by Onco*type* DX as “definitive”, contrasting with previous clinical assessments of her tumour. Felicity had faced particular uncertainties around adjuvant chemotherapy having received hormone treatment prior to surgery, and had found chemotherapy decision-making “impossible”. She welcomed the Onco*type* DX result, which squarely placed her at high risk, providing a clear recommendation for chemotherapy. For these participants, the fact that the test result directed treatment decision-making in this way distinguished it from previous layers. As well as being “definitive” in the sense of not able to be technically surpassed by other techniques (“state of the art”), the perceived finality of the Onco*type* DX result can also be linked to the fact that it directly recommended a treatment. Due to their fears surrounding recurrence, but also of chemotherapy, the discernment of the ‘right’ treatment was an important outcome of the diagnostic process for interviewees. For the participants above, by successfully resolving uncertainties around treatment, no further ‘layers’ of diagnostic information were needed. Bethany, who earlier likened breast cancer diagnosis to a ‘process of elimination’, described the test as a ‘final diagnosis’:

It just really meant that it was a very individualised, you know, that it was another layer, a bit like peeling an onion until you get to the final bit, you know, the final sort of diagnosis really.

Here Bethany points to an alternative conceptualisation of diagnostic layering, where various components of information are gradually uncovered until the most refined is provided by Onco*type* DX. Importantly, Bethany discussed this information as a ‘final’ layer because, due to the fact it was “individualised”, she could now make a decision about chemotherapy. Complementing Dette's earlier description of the diagnostic process thus far, Julie similarly proclaimed that the test acted as “a bit of a crystal ball, you know, and g[a]ve me something to actually make a decision on”.

In the context of uncertainty surrounding a feared treatment, Onco*type* DX was therefore welcomed by many participants interviewed for this research. The test was seen to solidify diagnoses which had previously been experienced as fluid and changeable. It ‘finalised’ diagnosis in two ways; through its provision of ‘state of the art’ and personalised diagnostic information, which could not technically be surpassed, but also by clearly directing treatment, ending uncertainties surrounding this important aspect of their cancer care. Nevertheless, receiving the Onco*type* DX score did not straightforwardly finalise the diagnostic process for every patient. As we now discuss, the introduction of the technique also had the potential to further unsettle paths to treatment, particularly in terms of its blurring of diagnosis and prognosis.

### 4.2 Meanings of gene expression profiling and the persistence of provisionality

As discussed in the first part of our findings, diagnostic information derived from established tools had the potential to reconfigure initially ‘good’ cancers as potentially ‘bad’ cancers. This rendered initial diagnoses as incomplete and mutable, with associated treatment plans experienced as a series of ‘false horizons’ (Hazel). Novel genomic techniques such as Onco*type* DX are presented as bringing finality, with clinicians often presenting such techniques as “the most comprehensive test” (Timmermans et al., 2017: 442). However, genomic information can sometimes introduce unexpected or contradictory results, as we heard during our interviews, causing confusion for patients and further disrupting diagnostic journeys.

In some cases, our interviewees’ Onco*type* DX results conflicted with previous layers of diagnostic information. Where these contradictions arose, unlike in the extracts above the genomic result was not always taken at face value, but required further interpretation. During the earlier stages of diagnosis, Lillian had been told “on two or three occasions” that hers was a “low risk cancer”. However, Onco*type* DX indicated that chemotherapy would be recommended for her. Having learned her result she decided that chemotherapy was “inevitable”, and welcomed the presentation of statistics and percentages alongside her score to help her make her decision. However, she also felt confused about her result, a feeling which lingered beyond her consultation:

It's only when I got home and I looked at my notes, I almost couldn't marry the two things together. Him saying it's low risk but then me being a 29 on the Onco*type*.

This was also the case for Dette, for whom the possibility of chemotherapy first arose following surgery. Despite being consistently advised that her cancer was “small” and “early”, her Onco*type* DX score placed her in the ‘high risk’ category. She discussed her confusion about this with her oncologist:

‘You don't have to be terrified’ [the oncologist] said, ‘you, your prognosis is incredibly good.’ And this is what's difficult to understand, is why give me chemotherapy? And she said ‘it's just because we don't want you to get it again.’

In these women's reflections, their cancer had been positioned as ‘good’ based on previous layers of information, but rendered a ‘high risk’ cancer by the Onco*type* DX test. This apparent contradiction is mitigated by the oncologist above through her distinction between Dette's cancer in the present (which had been successfully excised), and a possible cancer recurrence in the future. This demonstrates that the ambiguity of Onco*type* DX as a prognostic or diagnostic tool, as well as having implications for practitioners (Bourret et al., 2011), can impact patients. All of those we interviewed emphasised the value of Onco*type* DX as related to its implications for chemotherapy decision-making, an exercise most often associated with diagnosis and generally situated in the present. This is inscribed within the very name of the test itself, through use of the abbreviation *DX*. However, gene expression profiling differed from earlier assessments of their cancer, by drawing on predictive information. Where this information did not correspond, it unsettled patients such as Dette and Lillian. The significance of this for sociological studies of genomic technologies is that though often presented as a most “advanced” and “comprehensive” technique (Timmermans et al., 2017: 442), in these examples genomic testing did not render previous layers of diagnostic information obsolete. Despite a clear indication for treatment, Dette sought further discussion of her Onco*type* DX score in light of previous results. These became enmeshed in her interpretation of the score and its implications for treatment, with their significance not diminished by this novel technique.

This was also the case for Wendy and Zoe. They both consulted with their oncologist to further interpret the meaning of Onco*type* DX for treatment, despite both attaining a clearly low score. Following pathology results after a second surgery, Zoe had interpreted that her cancer was ‘high risk’ due to it being a high grade. She raised this with her oncologist:

I was a bit worried with a high grade on its own, it would be an indication for, for chemo, and, um. So I didn't, I didn't want to appear that I wanted chemo but I just wanted to make sure that not having it is the right thing to do. And, um, so she said, ‘no, no, no, at score 7 we would definitely not offer you chemo’.

Zoe's involvement of the oncologist in translating the Onco*type* DX result into a treatment decision, despite a clearly ‘low risk’ result, was shaped by a persistent unease about declining chemotherapy, in the context of a previous layer of diagnostic information which had labelled her cancer as ‘high grade’. She found it difficult to unequivocally accept the invalidation of this earlier information by the test, being unsure as to whether forgoing chemotherapy was “the right thing to do”. Zoe implies a felt imperative to embark upon chemotherapy to successfully treat cancer, despite her wish to avoid the treatment. This was also alluded to by Dette who discussed the sociocultural entwinement of cancer and chemotherapy: “you've got cancer, it's like “oh my God!” And everybody knows that chemotherapy gets rid of it”. This felt imperative was also invoked by a clinician in an appointment we observed, where an ‘intermediate’ Onco*type* DX result was discussed with a patient.

[The consultant] reiterated that it’s difficult for him to advise [on chemotherapy] one way or another. But he added ‘what if’ seems to be a ‘powerful driver’, i.e. ‘doing everything I can’ mentally. The patient said ‘it's no-brainer for me then’. **Observation2 June 2017**

In these examples, where the result disrupted prior configurations of their cancer, the information provided by Onco*type* DX is presented a *not quite* representing a ‘final’ layer of diagnosis, but as requiring further interpretation. Dette, Zoe and Wendy therefore sought further discussion with their clinicians about the meaning of their Onco*type* DX result, despite it providing a clear indication for treatment. As we have shown, their reasons were twofold. First, the result had contradicted previous layers of diagnostic information about their cancer. This demonstrates that all diagnostic layers in their totality remained important to participants, and were not simply surpassed by the information derived from genomic testing. Secondly, persistent fears about cancer and its possible recurrence meant that even in the context of a ‘low risk’ result, some reported anxieties about foregoing chemotherapy, despite this clear recommendation from an “advanced” test. Though previously unattainable, following Onco*type* DX testing Dette, Zoe and Wendy were now given an explicit treatment recommendation by their clinician. For the patient in Observation2 June 2017, this remained elusive following an intermediate result, though a recommendation to proceed to chemotherapy was subtly implied by her oncologist. Importantly, this was not solely formulated based on his assessment of layers of diagnostic information, but also by drawing on the powerful discourse of ‘doing all one can’ to fight cancer.

In these sections, we have observed some clinicians and participants discussing Onco*type* DX results as remedying the partial and mutable experiences of diagnosis hitherto described by interviewees. However, not all interviewees represented the test in this way. For some, despite patient and clinician anticipations for genomic testing to bring finality to diagnostic journeys, the result was not depicted as definitive. Importantly, our analysis has shown that despite its imagined finality and superiority to other techniques, decision-making following Onco*type* DX testing did not always incorporate the result unquestioningly. Instead, engagement with the result was complex and cautious, shaped by configurations of the tumour developed through previous diagnostic layers, as well as persistent fears of cancer and its possible recurrence.

## 5 Discussion

Breast cancer is now widely viewed as a heterogeneous disease within clinical settings. We have outlined, from a patient perspective, the routine processes through which breast cancer comes to be split into distinct subtypes. Within the UK NHS, the subtyping of breast cancer has given rise to different therapeutic regimens for individual patients, with these intersected by varying patterns of surgical management according to the cancer's size and spread. Breast cancer subtyping has become more refined, with an aim to ‘split’ diagnostic classifications falling under the ‘lumped’ category of breast cancer. The process of splitting a diagnosis of breast cancer entails several techniques, some well-established and others introduced only within the last decade. These developments have had consequences for patients as their tumour tissue becomes subjected to an increased number of diagnostic procedures, and as diagnostic information is delivered to them at several time-points. We have introduced the concept of ‘diagnostic layering’ to convey this contemporary experience of diagnosis as articulated by patients.

As a concept, layering captures both the incompleteness of diagnosis, experienced as partial and unable to be confirmed until further layers of diagnostic information are attained, and also the mutability of diagnosis, which could become subject to change as new layers of diagnostic information are introduced. Shifts in understandings of their cancer could be challenging for those participants whose subtype had initially been portrayed as unequivocally ‘good’, as this became invalidated by, for example, a designation of a higher grade. Despite patient hopes for it to resolve these uncertainties, the results of Onco*type* DX testing could further unsettle previous diagnoses, shifting patients' expected outcomes and treatment pathways. For our participants, routine diagnosis thus entailed contradictions, and periods of navigating a ‘diagnostic limbo’ (Nettleton, 2006), likening experiences of early breast cancer to those of contested illnesses (Purkiss and Van Mosell, 2008). Interviewees gave descriptions of managing these uncertainties emotionally by anticipating ‘false horizons’, ‘preparing for the worst’, and taking their cancer care ‘one step at a time’.

For our participants, the partial and protracted nature of diagnosis could be especially difficult to manage due to its direct implications for treatment; most notably adjuvant chemotherapy. Previous studies have shown how the ‘tangled web’ of 21st century oncology can blur prognosis, diagnosis and treatment from the perspective of practitioners, with implications for professional jurisdictions (Bourret et al., 2011; Keating and Cambrosio, 2013). We have shown the implications of reconfigurations of these practices for patients, as they attempt to make sense of their cancer (risk) when assessed by a range of diverse and sometimes inconsistent clinical tools, deployed at various time-points. As a relatively recent addition to routine diagnostic processes in the UK, Onco*type* DX testing is often touted as bringing certainty and closure to diagnosis in early breast cancer, particularly as it relates to treatment. Indeed, some participants' experiences did accord with this narrative. Some described the technique as ‘finalising’ diagnosis, in part because it was viewed as ‘state of the art’ and ‘personalised’ when compared with previous diagnostic tools they'd encountered. This inspired confidence in its recommendation. Its finality was also related to the fact that in the face of an agonising decision, its result could provide a clear recommendation for treatment. In these cases Onco*type* DX testing might be understood in similar ways to the numerical biomarker tests described by Bell (2013), whereby in the context of “cancer's semiotic ‘din’“, numerical results can provide a “reassuringly concrete buoy for patients to cling to” (p 134–5).

However, gene expression profiling also had the potential to extend the diagnostic process. This accords with research pointing to the creation and exacerbation of uncertainty resulting from novel genomic techniques (Kerr et al., 2019; Skinner et al., 2016; Timmermans et al., 2017). In some cases, participants returned to clinical judgement to interpret the implications of their gene expression test result for treatment. This was not only in the case of an intermediate score, a ‘zone of uncertainty’ where clinical judgement is more often called upon (Bourgain et al., 2020), but also observed in cases where a recommended course of action was clearly inscribed within the result. Patient deferral to their clinician points to a “persistence of the clinic” (Latimer et al., 2006: 623), despite suggestions that genomic technologies would relegate clinical judgement. In such cases, clinicians weaved together sometimes contradictory diagnostic layers, patient priorities, concerns and fears, and professional assessments with this genomic information, in order to develop an appropriate treatment recommendation for their patient. Of note in this research is that clinical judgement also drew on cultural narratives entwining chemotherapy and cancer, and the possibility of future regret.

The layer of diagnostic information provided by Onco*type* DX, despite often being represented as advanced, and experienced as such by some patients, in fact shared many characteristics with that provided by established tools. Due to its conflation of diagnostic and prognostic information, like previous diagnostic layers the Onco*type* DX result could contradict established configurations of patients' cancers, causing confusion and anxiety. Our analysis has shown that despite its purported exceptionalism, the result was not interpreted in isolation by patients, but made sense of in light of previous diagnostic results. Their significance was not reconfigured by the technique. Further, like previous layers the Onco*type* DX score remained open to contestation and re-interpretation, with some participants embarking upon further discussions with their clinician about its meaning, even in apparently clear-cut cases. Lastly, though marked as distinct by some interviewees for providing ‘scientific facts’ on which to base a treatment decision, interpretations of the test result remained couched within persistent fears of cancer and its possible return. These influenced the way all participants engaged with Onco*type* DX, provoking strong emotional engagement with results, whether these indicated chemotherapy or not.

## 6 Conclusion

Our research has attended to routinized and everyday practices of breast cancer diagnosis in the UK, into which molecular examination has been embedded for many years. We have contributed to the sociology of diagnosis by attending in-depth to how contemporary techniques that ‘split’ breast cancer into subtypes are experienced by patients. Our analysis has demonstrated that multiple laboratory examinations of tumour tissue extend diagnosis for patients, and introduce more opportunities for the invalidation of women's anticipations for their treatment and care. The concept of diagnostic layering, directly developed from patient accounts, captures these experiences of contemporary breast cancer diagnosis as partial and incomplete, and as mutable and subject to change.

As a relatively recent introduction to routine diagnostic processes, Onco*type* DX could be represented as a ‘final’ diagnostic layer, due to its ability to determine treatment where previous techniques had failed. Patients anticipated that the technique would resolve treatment uncertainties provoked by established diagnostic tools, and in some cases take the heavy responsibility for treatment decision-making out of their hands. We have shown that for some patients this was the case, with these women attributing their “confidence” in this genomic test to its “advanced” nature, and its provision of a direct recommendation for treatment. However, our analysis has also highlighted that the result did not straightforwardly direct treatment decisions for all. Some women described the incorporation of the result into their decision-making as informed by diagnostic information arising from well-established tools, which could entail contradictions, and as powerfully shaped by fears of cancer and its return. This led several participants to interrogate the meaning of the result further, requiring additional interpretation and disrupting anticipations for the finalisation of diagnostic and treatment pathways. In these cases the enduring significance of the clinician in interpreting genomic information, and translating this into a treatment recommendation for the individual patient, was clear.

In line with personalised medicine's aim to further refine diagnosis and treatment, it is likely that additional layers of diagnostic information derived from novel genomic techniques will become incorporated into routine cancer care. However, scientists now highlight the diversity of breast cancer even within individual patients, and as shifting over time (Martelotto et al., 2014). This calls into question the extent to which the much sought after ‘finality’ in cancer diagnosis can be achieved, with layering perhaps better understood as a process continuing even beyond treatment. Our article has highlighted the importance of sociological attention to the specificities and temporality of diagnostic layers; how they are produced and in what contexts, and of the lived consequences of these diverse forms of clinical information for patients. Our analysis of patient accounts of Onco*type* DX has shown that patients make sense of novel forms of diagnostic information in terms of the techniques that have produced it, the extent to which it aligns with other sources of diagnostic information, and in the context of wider disease experiences. Such insights are important for medical practice, as patients face navigating further layers of complex diagnostic information, and as clinicians manage patient (as well as their own) expectations for genomic techniques to resolve diagnostic uncertainties (Kerr et al., 2019).

## Author contribution

ER developed the idea for this piece and wrote the article with JS. ER and CKC led qualitative data collection. SCB and AK devised the wider research project and secured funding. All authors were involved in developing and editing this article.

## Declaration of competing interest

We have no conflicts of interest to disclose.
